# Automatic identification of myopic maculopathy related imaging features in optic disc region via machine learning methods

**DOI:** 10.1186/s12967-021-02818-1

**Published:** 2021-04-26

**Authors:** Yuchen Du, Qiuying Chen, Ying Fan, Jianfeng Zhu, Jiangnan He, Haidong Zou, Dazhen Sun, Bowen Xin, David Feng, Michael Fulham, Xiuiyng Wang, Lisheng Wang, Xun Xu

**Affiliations:** 1 The Institute of Image Processing and Pattern Recognition, Department of Automation, Shanghai Jiao Tong University (SJTU), 800 Dongchuan RD. Minhang District, Shanghai, 200240 People’s Republic of China; 2 Department of Preventative Ophthalmology, Shanghai Eye Diseases Prevention and Treatment Center, Shanghai Eye Hospital, No. 380 Kangding Road, Shanghai, 200040 China; 3 Department of Ophthalmology, Shanghai Key Laboratory of Ocular Fundus Diseases, Shanghai Engineering Center for Visual Science and Photo Medicine, Shanghai General Hospital, SJTU School of Medicine, Shanghai, China; 4 National Clinical Research Center for Eye Diseases, Shanghai, 20080 China; 5 Biomedical and Multimedia Information Technology Research Group, School of Computer Science, The University of Sydney, Sydney, NSW 2006 Australia; 6 Department of Molecular Imaging, Royal Prince Alfred Hospital and the University of Sydney, Sydney, Australia

**Keywords:** Pathologic myopia, Myopic maculopathy, Feature mining, Machine learning, Radiomics

## Abstract

**Background:**

Myopic maculopathy (MM) is the most serious and irreversible complication of pathologic myopia, which is a major cause of visual impairment and blindness. Clinic proposed limited number of factors related to MM. To explore additional features strongly related with MM from optic disc region, we employ a machine learning based radiomics analysis method, which could explore and quantify more hidden or imperceptible MM-related features to the naked eyes and contribute to a more comprehensive understanding of MM and therefore may assist to distinguish the high-risk population in an early stage.

**Methods:**

A total of 457 eyes (313 patients) were enrolled and were divided into severe MM group and without severe MM group. Radiomics analysis was applied to depict features significantly correlated with severe MM from optic disc region. Receiver Operating Characteristic were used to evaluate these features’ performance of classifying severe MM.

**Results:**

Eight new MM-related image features were discovered from the optic disc region, which described the shapes, textural patterns and intensity distributions of optic disc region. Compared with clinically reported MM-related features, these newly discovered features exhibited better abilities on severe MM classification. And the mean values of most features were markedly changed between patients with peripapillary diffuse chorioretinal atrophy (PDCA) and macular diffuse chorioretinal atrophy (MDCA).

**Conclusions:**

Machine learning and radiomics method are useful tools for mining more MM-related features from the optic disc region, by which complex or even hidden MM-related features can be discovered and decoded. In this paper, eight new MM-related image features were found, which would be useful for further quantitative study of MM-progression. As a nontrivial byproduct, marked changes between PDCA and MDCA was discovered by both new image features and clinic features.

**Supplementary Information:**

The online version contains supplementary material available at 10.1186/s12967-021-02818-1.

## Background

Pathologic myopia is a major cause of visual impairment and blindness worldwide, especially in East Asian populations [1, 2]. Mostly developing from high myopia, pathologic myopia may cause a wide spectrum of complications, such as glaucoma, retinal detachment, and myopic maculopathy. Myopic maculopathy (MM), also known as myopic macular degeneration, is the most serious and irreversible complication. It is estimated that by 2050, visual impairment from MM will affect 55.7 million people and 18.5 million will become blind [3]. According to the International Photographic Classification and Grading System for myopic maculopathy (META-PM) [4], MM from mild to severe could be graded as no macular lesions (C0), tessellated fundus (C1), diffuse chorioretinal atrophy (C2), patchy chorioretinal atrophy (C3), and macular atrophy (C4). A higher grade of MM has more marked fundus changes and visual impairment. Thus, the identification of early fundus changes in patients who then further develop MM may offer the potential insights for earlier intervention and reduction in visual impairment and blindness.

The early identification of MM, however, is challenging. Fang et al. [5] and Yan et al. [6] recently reported that the peripapillary atrophy (PPA) was a risk factor for the development from high myopia to pathologic myopia and the development of MM. Our previous study[7] also suggested that tilt ratio and PPA area were associated with macular and peripapillary choroidal thickness in young high myopic patients. These studies indicated that the region of the optic disc has features that may correlate with the development of MM. In the short term, these features could imply different fundus image changes between population who develop severe MM and who do not. In a long run, because optic disc changes usually occur in high myopia stage, these features also have potential to serve as indicators to discern the high-risk population in an early stage. Thus, exploring and quantifying more hidden or imperceptible MM-related features to the naked eyes in optic disc region may contribute to a more comprehensive understanding of MM and therefore may assist to distinguish the high-risk population.

Radiomics is a methodology that extracts a large number of features from medical images using data-characterization algorithms. These features offer potential to discover disease characteristics that are beyond the perception capacity of the naked eye. Although not yet used in ophthalmic imaging analysis, radiomics has been widely used in tumor phenotype classification, preoperative prediction, treatment assessment and etc. based on image feature mining [8–17]. Recently, machine-learning has been used to identify image features in a variety of eye diseases including diabetic retinopathy, glaucoma and macular degeneration [18–21]. However, the morphological assessment of the optic disc region in pathologic myopia is complicated, and the features solely from surface are far from sufficient for more accurate diagnosis and more descriptive characteristics are yet to be revealed. Employing machine learning and radiomics methods to extract and quantify these hidden image features could help discover more MM-related fundus image characteristics, and transfer retinal specialists’ experience to practical evaluating indicator to help make diagnosis [22].

This research aims to (1) mine MM-related image features from the optic disc region of fundus images via machine learning and radiomics methods; and (2) provide quantitative analysis on MM characteristics to assist ophthalmologists have further insights of the development of MM, and to support objective early diagnosis.

## Patients and methods

### Patients

The patients included in this study were recruited in the Shanghai High Myopia Study for Adults. The study protocol has been described in our previous study [23] and was approved by the Medical Ethics Committee of Shanghai General Hospital, Shanghai Jiao Tong University School of Medicine, consistent with the Declaration of Helsinki. Written informed consent was obtained from all study participants. The study (# NCT03446300) was registered at http://www.clinicaltrials.gov.

They were over 50 years of age and had an Axial length (AL) more than 26 mm. Exclusion criteria included: an IOP over 21 mmHg; previous intraocular or refractive surgery other than cataract surgery; media opacity; coexisting or history of ocular or severe systemic diseases, including dense cataract, glaucoma, diabetic retinopathy or diabetic macular edema and autoimmune disease; and other evidence of retinal pathology not related to myopia. Patients were also excluded if the retinal images were ‘poor’, for example, images out of focus, interfered of light, occluded and etc. Generalized estimating equation regression models were used to account for correlation between left and right eyes of the same patient [23]. Both eyes were included in this study, and if an eye was not eligible based on the inclusion criteria, it was excluded from this particular analysis.

According to the META-PM Study Group [4], myopic maculopathy was classified into C0, C1, C2, C3, and C4. According to the earlier reports, [5, 24–26] C2 was sub-classified into peripapillary chorioretinal atrophy (PDCA) and macular chorioretinal atrophy (MDCA). Lacqure cracks, myopic choroidal neovascularization and Fuch’s spot were classified as “plus lesions”. Eyes with graded greater than or equal to MDCA or associated with plus lesions were classified as severe MM group, while eyes with C0, C1 or PDCA were classified as without severe MM group. The classification and grading of MM were performed by two independent, well-trained graders (Q.Y.C and J.N.H). In cases of disagreement, an adjudication was made by a retinal specialist (Y.F). The clinical characteristics of the patients are listed in Table 1.

**Table 1:** Patient clinical characteristics

	Total	C0	C1	PDCA	MDCA	C3	C4	*P* value^a^	Without severe Myopic Maculopathy (MM)	Severe MM	*P* value^b^
Eye # (%)	457	44(9.6)	112(24.5)	122(26.7)	104(22.8)	45(9.8)	30(6.6)	/	278	179	/
Age in yrs	67.9 ± 6.3	64.6 ± 5.7	67.6 ± 5.2	67.4 ± 6.1	69.1 ± 7	69 ± 6.3	70.5 ± 7.6	< 0.001	67 ± 5.7	69.3 ± 6.9	< 0.001
Men (%)	191(41.8)	27(61.4)	47(42.0)	54(44.3)	42(40.4)	13(28.9)	8(26.7)	0.021	128(46.0)	63(35.2)	0.025
SER, D	− 9.34 ± 5.65	− 6.17 ± 3.09	− 8.27 ± 4.85	− 8.9 ± 4.82	− 11.83 ± 6.34	− 9.88 ± 6.7	− 10.28 ± 6.8	< 0.001	− 8.21 ± 4.68	− 11.08 ± 6.53	< 0.001
AL, mm	28.53 ± 1.85	26.71 ± 0.53	27.62 ± 1.37	28.18 ± 1.65	29.62 ± 1.68	30.26 ± 1.4	29.63 ± 1.74	< 0.001	27.72 ± 1.49	29.79 ± 1.64	< 0.001
BCVA (logMAR)	0.46 ± 0.37	0.16 ± 0.14	0.35 ± 0.29	0.42 ± 0.34	0.6 ± 0.38	0.59 ± 0.41	0.95 ± 0.35	< 0.001	0.35 ± 0.31	0.64 ± 0.4	< 0.001
Tilt ratio	0.70 ± 0.13	0.78 ± 0.1	0.7 ± 0.14	0.72 ± 0.12	0.65 ± 0.13	0.63 ± 0.13	0.66 ± 0.11	< 0.001	0.72 ± 0.13	0.65 ± 0.13	< 0.001
Torsion^0^	− 2.89 ± 33.72	6.52 ± 46.43	− 5.43 ± 34.44	− 1.43 ± 41.04	− 5.35 ± 23.94	− 0.16 ± 20.67	− 8.76 ± 14.06	0.303	− 1.78 ± 39.52	− 4.62 ± 21.85	0.326
PPA area (mm^2^)	2.27 ± 2.18	0.52 ± 0.34	1.45 ± 1.16	1.53 ± 1.14	3.52 ± 2.59	4.15 ± 2.86	3.77 ± 2.12	< 0.001	1.34 ± 1.12	3.72 ± 2.59	< 0.001

*AL *axial length, *BCVA*   best-corrected visual acuity, *C0*  no myopic maculopathy; *C1*   tessellated fundus, *C3 * patchy atrophy, *C4*   macular atrophy, *D*  diopter, *logMAR*   logarithm of minimal angle of resolution, *MDCA*   macular diffuse choroidal atrophy, *PDCA*   peripapillary diffuse choroidal atrophy, *PPA*  peripapillary atrophy, *SER*  spherical equivalent refraction; Eyes with C0, C1 and PDCA were classified as without severe MM, eyes with MDCA, C3 and C4 were classified as severe MM

^a^*P* value for the difference in characteristics between different degree of myopic maculopathy, based on One-way ANOVA test or x^2^ test, as appropriate

^b^*P* value for the difference in characteristics between eyes with and without severe MM, based on Student’s *t*-test or x^2^ test, as appropriate

A total of 457 eyes of 313 participants with high myopia were included in the study, noted as the whole dataset. We split 70% of the eyes as training dataset consisting of 319 eyes, and 30% as validation dataset consisting of 138 eyes.

### Methods

The radiomics method involved: (1) delineation of the regions of interests (ROIs); (2) construction of a feature pool by the automatic extraction of image features from the ROIs and, (3) selection of discriminative features using the correlation analysis and machine learning method.

### ROI delineation

**Fig. 1:**
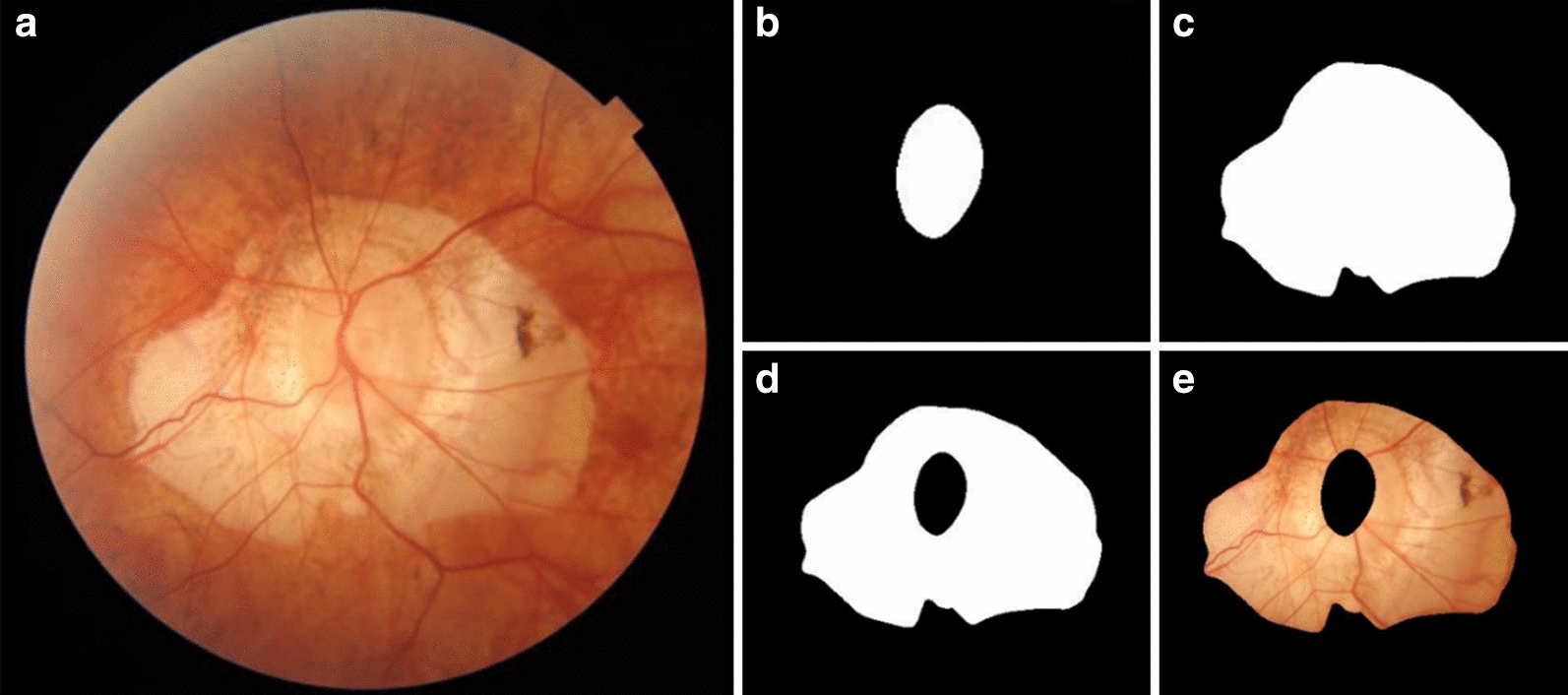
Delineation of ROI: **a** original fundus image; **b** mask of optic disc; **c** mask of contour of PPA with optic disc; **d** mask of PPA region and, **e** masked PPA

The ROIs were interactively extracted and annotated from fundus images according to the PPA and optic disc boundaries using an in-house annotation software as shown in Fig. 1. The contours of the delineated regions were smoothed through B-spline interpolation to avoid blurs generated by manual errors [27].

### Construction of the feature pool

Before the feature extraction, all scans of left eyes were mirrored to conform to scans of right eyes. There were 316 high throughput features that were extracted and comprised 151 morphologic, 54 intensity and 111 texture features. All of the features were extracted from the ROI of images (Fig. 1). Various morphological characteristics of the optic disc region included regional properties, such as the Hausdorff distance and the curvatures of contours, and global properties, such as perimeters, central moments and moment invariants [28, 29]. Corresponding shape description operators were used to obtain these features. The intensity features described intensity distributions, such as mean value, variance, and statistics histograms. The definition of intensity and texture features was detailed in the website (https://pyradiomics.readthedocs.io/en/latest/). These features were extracted with the PyRadiomics package from R, G, B channels of color fundus images, separately [30].

Six shape features had been reported previously by ophthalmologists and regarded as possible MM-related candidates [31–33]. We termed these six features as clinic features which conducted clinic feature set. Among them, AreaPPA was defined as the area between the contour of PPA and the contour of optic disc. AreaDisc was defined as the area within the contour of optic disc. Tilt of optic disc was defined as the ratio of the short axis and the long axis of optic disc. Torsion referred to the angle between the long axis of optic disc and the line perpendicular to the line of macular and center of optic disc. Dist_MD referred to the distance of macular and center of optic disc. Angle_MD referred to the angle between horizontal and line of macular and center of optic disc. All of the indexes were counted in pixels. Since all of the color fundus images had the same size of 2032*1934 with 150 dpi, it has been ensured that these data were obtained under the same metric. These six clinic features were included into shape features subclass for further selection.

### MM-related features selection

We selected MM-related features from 322 features based on evaluating their ability to classify patients with and without severe MM. The data in training dataset were normalized by Z-score in advance [34, 35]. First, mutual information and student’s t test were employed to filter out noisy and irrelevant features [36, 37]. Then, SFFS (Sequential Floating Forward Selection) algorithm with random forest classifier was used to select the optimal feature set [38, 39]. fivefold cross validation was employed to avoid over-fitting in SFFS procedure. Finally, a final feature set was selected according to the number of features, mean score and standard deviation of cross validation. A decision model was trained using this feature set by random forest classifier. ROC (Receiver Operating Characteristic) and AUC (Area Under the Curve) indexes were used to evaluate the feature set’s performance of classifying patients with or without severe MM.

### Statistical analysis

The final feature set was termed new feature set. The features in the final feature set were termed new image features. To assess how the new feature set would generalize an independent data set, the performance of decision model was evaluated on the validation dataset. To assess the effectiveness of each new image feature, the classification ability and intra-class distribution property of these features were calculated on the whole dataset. The classification ability of new image features was assessed by the univariate performance using the logistic regression model under fivefold cross validation. The intra-class distribution of new image features was illustrated by boxplot on patients with and without severe MM. To evaluate the independence of the new image features, the correlation between new image features and clinic features was evaluated using the Pearson Correlation Coefficient. To put the results, in visualization, we selected five high univariate score features (AUC > 0.75) from new image features and the clinic feature set and visualized them in graphs. Furthermore, we displayed the mean value and standard deviation value of the five features on subclasses of META-PM Study Group.

## Results and evaluation

The generalized estimating equation regression models revealed that there were no significant differences in ocular biometry between the two eyes; thus, no adjustment is needed for the associations between the two eyes. Therefore, the eyes, including those from a same person, were regarded as independent samples and were included in this study based on the inclusion criteria.

### Features selection results

**Fig. 2:**
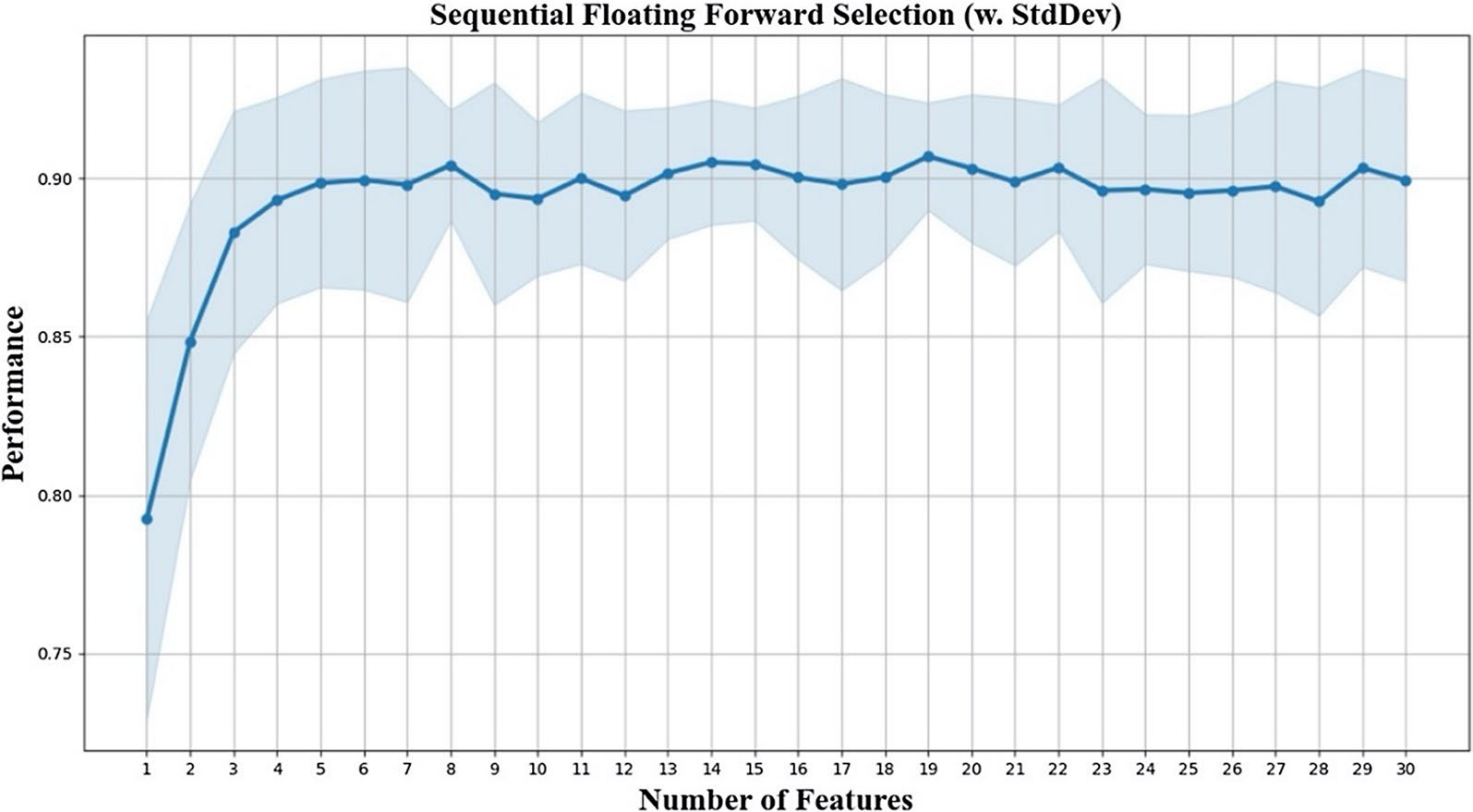
SFFS algorithm feature selection result; as the number of selected features increases, average scores and standard deviation of discriminative power on patients with and without severe MM fluctuated

Among the 322 features, 220 had passed mutual information filter (threshold = 0.2), and 244 had passed independent student’s t test (p < 0.05). An intersection of 188 features retained after the filter method phase. Among the clinic features, AreaPPA and Tilt retained while the others had been filtered out. The maximal number of features evaluated by SFFS was set to 30. The mean score of classification performance were arising until the number of selected features increased to eight (Fig. 2). For a better trade-off between the classification performance and the refinement of our model, we chose these eight features as the new feature set.

### Performance of new image features and clinic features

**Fig. 3:**
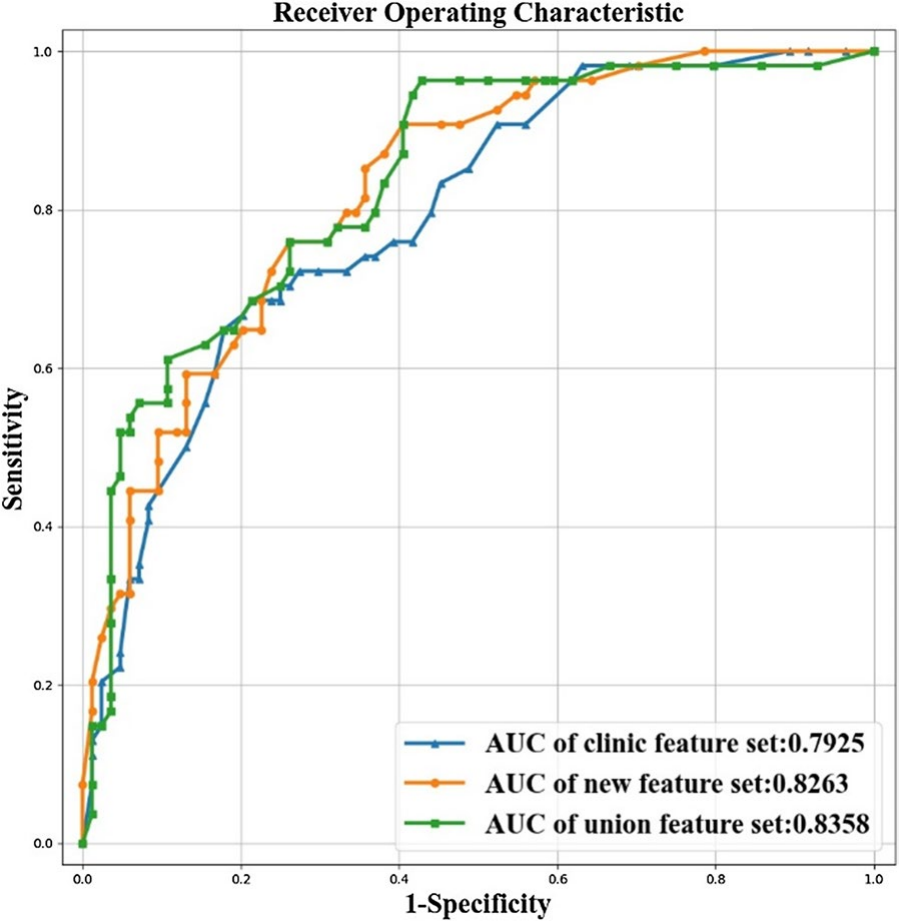
The ROC and AUC of new feature set, clinic feature set and union feature set on the validation dataset for classifying severe MM

The performance of new feature set in classifying severe MM was scored 0.8263 AUC on the validation dataset (Fig. 3). The performance of clinic feature set was scored 0.7925 AUC on validation dataset (Fig. 3). Finally, we combined the new image features and clinic features together to generate the union feature set. The union feature set got 0.8358 on validation dataset (Fig. 3).

The univariate performance of new image features and clinic features on the whole dataset as well as their definitions were listed in Table 2. Five new image features, including firstorder_Energy_R, firstorder_Entropy_B, Coarseness_NGTDM, PPAweight_u20R and glcm_shade_135, had the univariate AUC scores higher than 0.70. There was only one clinic feature, AreaPPA, had the individual AUC score higher than 0.70.

**Table 2:** New image features–description and properties

New image features			Clinic features		
FeatureIndex	Description	AUC	FeatureIndex	Description	AUC
Firstorder_Energy_R	The intensity distribution of PPA	0.8471 ± 0.0023	AreaPPA	The area of PPA	0.8317 ± 0.0226
Firstorder_Entropy_B	The intensity distribution of PPA	0.8369 ± 0.0377	Tilt	The tilt of optic disc	0.6742 ± 0.0620
Coarseness_NGTDM	The texture pattern of PPA	0.8176 ± 0.0243	Dist_MD	The distance between macula and optic disc	0.6013 ± 0.0259
PPAweight_u20R	The intensity distribution of PPA	0.7886 ± 0.0356	AreaDisc	The area of optic disc	0.5670 ± 0.0683
glcm_shade_135	The texture pattern of PPA	0.7085 ± 0.0339	Angle_MD	The angle of the horizonal and Dist_MD	0.5562 ± 0.0627
PPAweight_u30R	The intensity distribution of PPA	0.6727 ± 0.0807	Torsion	The torsion of optic disc	0.4649 ± 0.0362
Fourier_Circularity_PPA	The shape of PPA	0.6632 ± 0.0401			
DiscCurvature13	The shape of optic disc	0.6547 ± 0.0589			

### Statistics distribution of each feature on patients with and without severe MM

**Fig. 4:**
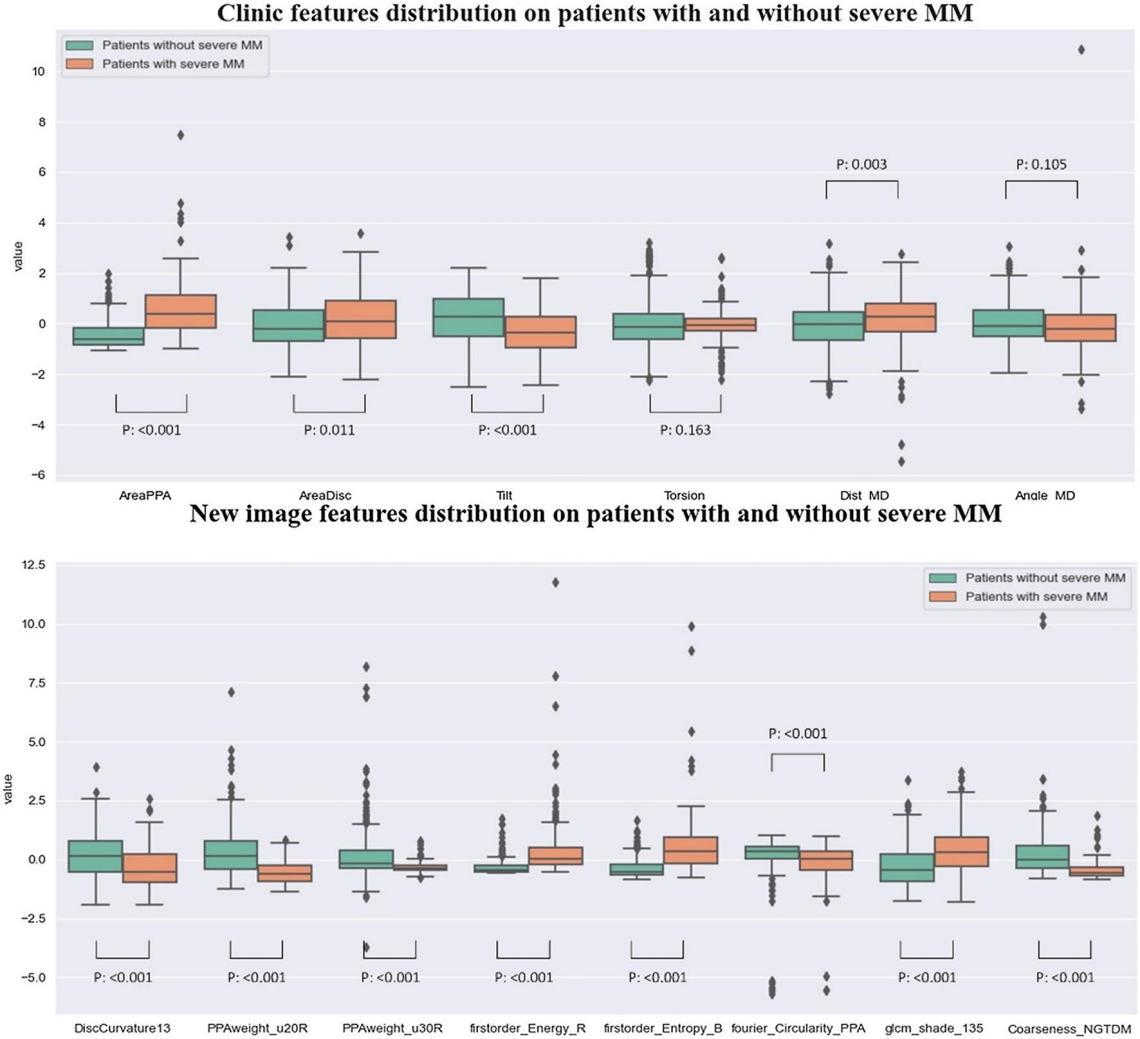
The boxplot of eight new image features and six clinic features

The feature selection phase has ensured the new image features had statistical discriminative power on patients with and without severe MM (student’s t test, p < 0.05). The boxplot figure has intuitively displayed the intra-class distribution of new image features and clinic features (Fig. 4) on patients with and without severe MM. All of the new image features and four clinic features (except for Torsion and Angle_MD) had significant differences between patients with and without severe MM (P < 0.05).

### Correlation between new image features and clinic features

**Fig. 5:**
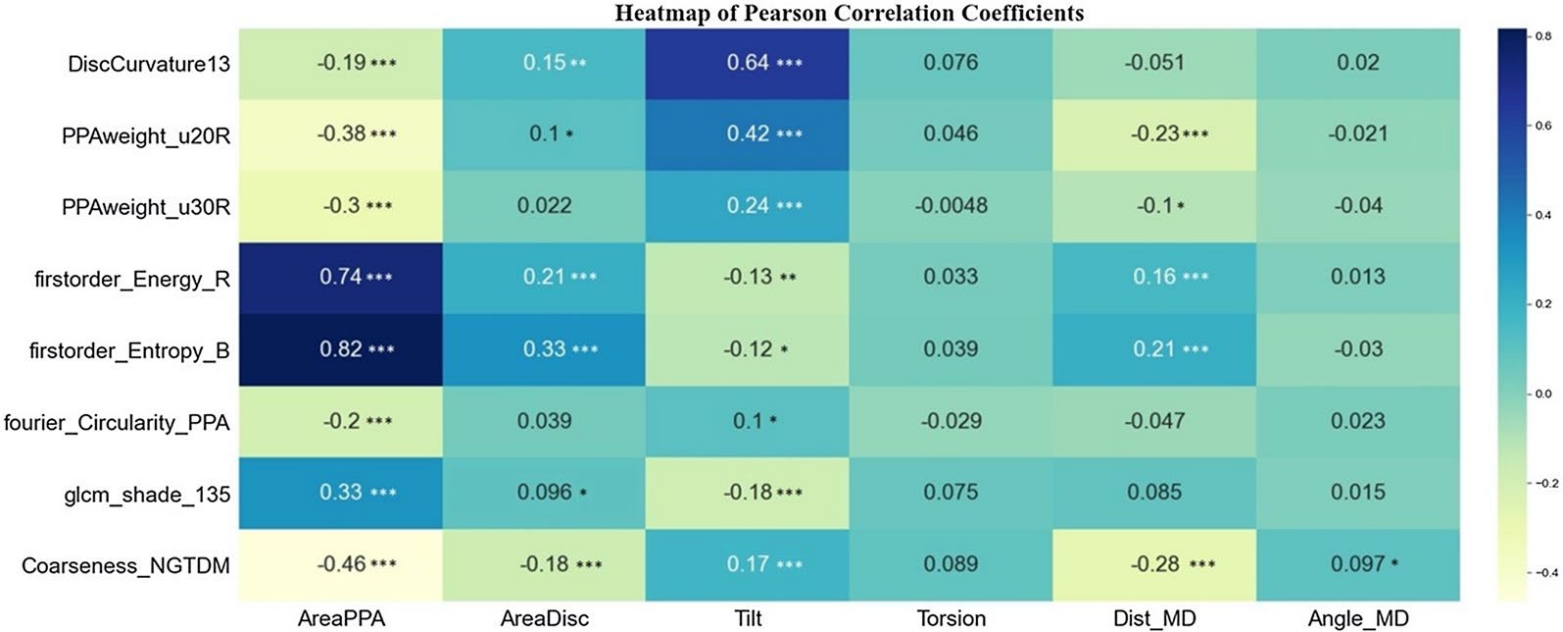
Correlation between new image features and clinical factors. (*p value < 0.05. **p value < 0.01. ***p value < 0.001. no marker: p value > 0.05)

The correlation coefficients between new image features and clinic features were quantitatively evaluated by Pearson Correlation Coefficient and shown in Fig. 5. Among the new image features, firstorder_Energy_R and firstorder_Entropy_B had high correlation coefficient scores with AreaPPA. The feature firstorder_Energy_R occupied the PPA area factors, so it was thought to be dependent with AreaPPA. The feature firstorder_Entropy_B depicted the entropy of the region rarely effected by area, so it was thought to be an independent factor. The other pairs of new image features had little relevancy with the clinic features. Thus, they were thought to be new findings.

### Visualization of new image features and clinic features

**Fig. 6:**
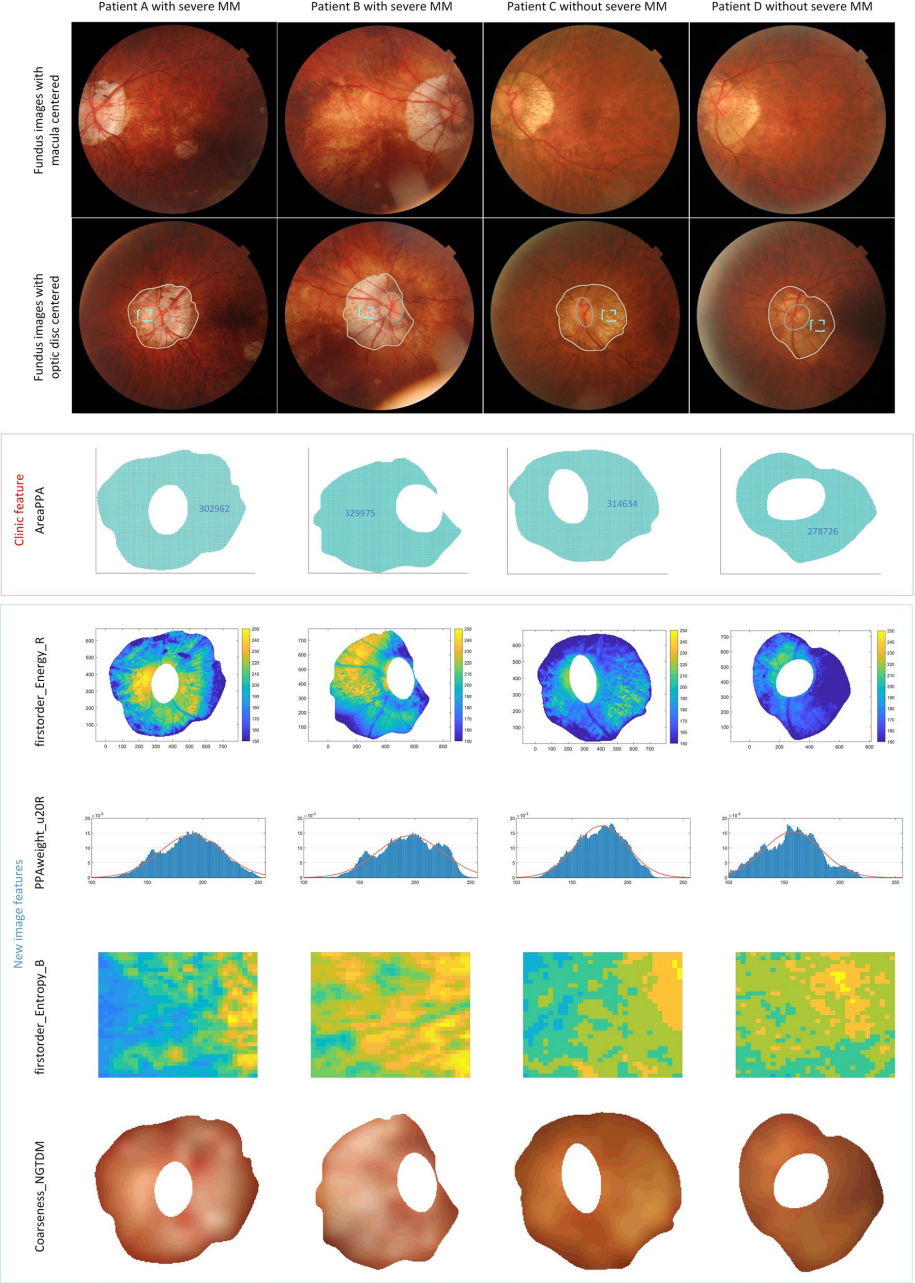
Visualization of new image features and clinic proposed features. Column 1 and 2: fundus images of two patients with severe MM. Column 3 and 4: fundus images of two patients without severe MM. The first row: the macula-centered fundus images. The second row: the corresponding optic disc-centered fundus images. The third row: AreaPPA (PPA areas. We chose patients with similar PPA areas as examples, only by counting areas could not distinguish if a patient had severe MM or not). The fourth row: firstorder_Energy_B (energy of PPA). Higher values of firstorder_Energy_B meant brighter intensity in PPA region. The fifth row: PPAweight_u20R (pixel distribution of PPA). Higher average values of PPAweight_u20R meant more rapid fluctuation of pixel values along broadwise direction of PPA region. The sixth row: firstorder_Entropy_B (entropy of PPA). Higher value of firstorder_Entropy_B meant more complexity pixel values distribution in PPA region (zoomed from the rectangular in row 2rd fundus images). The seventh row: Coarseness_NGTDM (Coarseness of PPA). Higher Coarseness_NGTDM values meant a lower spatial change rate and a locally less nonuniform texture

Four new image features (AUC > 0.75) and one clinic feature AreaPPA (AUC > 0.75) were visualized in four cases consisting of patients both with and without severe MM to help understanding (Fig. 6).

### Analysis of subclass of MM

**Fig. 7:**
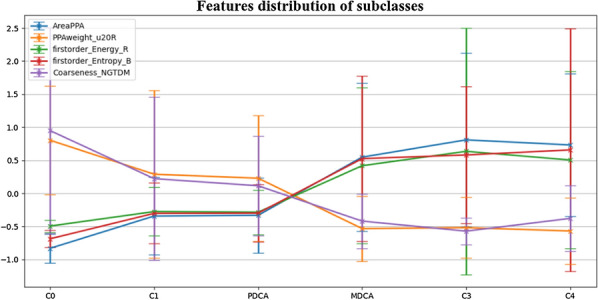
The mean values and standard deviation values of features changed among subclasses. Significant changes existed between C0 and C1, and PDCA and MDCA

In the whole data set, patients were divided into six subclasses: C0, C1, PDCA, MDCA, C3, C4. We explored how the values of MM-related features would change along with these different subclasses. For the four new image features (AUC > 0.75) and one clinic feature AreaPPA (AUC > 0.75), their mean values and standard deviation in each subclass were estimated on the whole data set (all subclasses had been labeled based on clinical reports), as shown in Fig. 7. It showed that the mean values of these features changed significantly at two places: one was between C0 and C1, another was between PDCA and MDCA.

## Discussion

In this study, we applied radiomics method into the field of ophthalmology to explore comprehensive MM-related features. Our main findings were as follows: (a) eight new image features were discovered to have better performance than clinic features in classifying severe MM patients. These features were difficult to be perceived in clinic; (b) an effective machine learning tool for automatically extracting and decoding various MM-related image features was developed; (c) marked changes between PDCA and MDCA was discovered by both new image features and clinic features.

We have found eight new image features based on their performances for classifying patients with and without severe MM. The overall performance of the new feature set was higher than the clinic feature set. The highest score was achieved from the combination of these two sets, which implied that the new image features revealed more characteristics related to MM and had complementary values to clinic features. Supporting cases were shown in Fig. 6. The new image features enabled the right diagnosis to complex cases, while in comparison the conventional clinical factor of PPA area failed.

Most of the new image features were new findings. Among them, seven out of eight were completely new factors by pearson correlation analysis, while the only common factor was relevant to the area of PPA. This common factor consistently supported the previous study results that PPA area was a risk factor for the development of MM. The seven new image features indicated that changes of intensity and texture also occurred in optic disc region between patients with and without MM in the cohort. Specifically, the feature firstorder_Energy_R indicated that the PPA of MM cases were bigger and brighter than those without MM in this study. The feature firstorder_Entropy_B indicated that the color level of PPA of MM cases was denser. And the feature Coarseness_NGTDM indicated the PPA of MM cases had locally more non-uniform texture. The highest performance of new features was 0.8471 AUC, implying comparability to the classic indicator PPA area (0.8317 AUC).

The new image features provided a more comprehensive view for the characteristics of the development of MM. The intensity, texture and high order morphology properties revealed by those features could only be sensuously perceived but difficult to interpret by routine clinic. However, this subjective operator-dependent expertise and experience may often play critical role on making accurate diagnosis for complex cases. With the capacity of providing quantitative and descriptive imaging characteristics of MM status, our study thus would serve to transfer the experience of retinal specialists to practical indicators, which therefore might inspire standardizing the diagnosis.

Moreover, the feature extraction and screening approach in this study was highly automatic. Previously, several optic disc morphologic changes were reported to be significantly associated with the progression of high myopia and the development and progression of MM [5, 6, 31–33]. However, these clinic features were measured manually and separately, and highly depended on the ophthalmologists’ experience. In addition, with difficulty to access the retinal specialists, junior ophthalmologists lacking of experience might not always be able to make correct clinical decisions. In this research, the fundus images were only required to be annotated for ROI regions in advance. High throughput features were extracted in automatic processing manner via computer-aided technique and MM-related features were filtered out automatically via an AI model. Our approach illuminated capability for rapid population-based screening, by providing time- and labor- efficient, and objective and quantitative measurement.

Another interesting finding in the present study was that, when analyzing changes in mean values of new image features and clinic features between different subclasses, most of the features changed remarkably in two places. One was from C0 to C1, another was from PDCA to MDCA. The substantial change from C0 to C1 was not hard to understand and mainly because in C0 there normally no fundus changes, while in contrast, C1 tended to have tessellated fundus. Interestingly and surprisingly, our study found that there was also a distinctive variation from PDCA to MDCA among most features, including both new image features and clinic features; and in comparison, the changes from MDCA to C3 and C4 were relatively slight. To avoid the possibility that the variation of features from PDCA to MDCA might be a common result that they were separately categorized, we additionally categorized them into the same group and repeated our experiments. The new selected features also illustrated large variations from PDCA to MDCA (Please refer to the Additional file 1). Previous studies classified PDCA, MDCA and more serious grades as MM [4, 40].

Our study suggested that MM could be further subdivided. For patients with PDCA, the fundus changes were mainly around the optic disc, and no significant lesions were found in the macular region. Thereby, it was more appropriate to classify PDCA as mild MM. It was reported that patients with PDCA had thicker macular choroidal thickness and better BCVA than patients with MDCA and severer MM in elder patients [24]. However, whether the elder patients with PDCA would progress to MDCA had not been reported in the literature. Hence, MM might to be further subdivided so that PDCA would be classified as mild MM while MDCA along with more serious grades defined as severe MM for the elderly population. This suggested further classification might be helpful for prognostic evaluation and follow-up plan guidance for different age groups. In the future study, we would collect follow-up data to further test our hypothesis.

## Conclusions

In conclusion, this study was the first effort on employing radiomics method to precisely quantify MM-related features that otherwise were difficult to be perceived in the routine clinic. And it was the first time to potentially substitute the subjective evaluation factors for the characteristics of different grades of MM based on retinal specialists’ experience with the objective and quantitative evaluation system. In the future, more follow-up studies are needed to prove that AI could help realize MM early prediction.

## Supplementary Information

**Additional file 1:** Supplementary Material.

## Data Availability

The data that support the findings of this study are available from Shanghai General Hospital but restrictions apply to the availability of these data, which were used under license for the current study, and so are not publicly available. Data are however available from the authors upon reasonable request and with permission of Shanghai General Hospital.

## Abbreviations

MM: Myopic maculopathy
PDCA: Peripapillary diffuse chorioretinal atrophy
MDCA: Macular diffuse chorioretinal atrophy
META-PM: International Photographic Classification and Grading System for myopic maculopathy
C0: No macular lesions
C1: Tessellated fundus
C2: Diffuse chorioretinal atrophy
C3: Patchy chorioretinal atrophy
C4: Macular atrophy
PPA: Peripapillary atrophy
ROI: Regions of interest
SFFS: Sequential Floating Forward Selection
ROC: Receiver Operating Characteristic
AUC: Area Under the Curve
