# Correction to: Automatic identification of myopic maculopathy related imaging features in optic disc region via machine learning methods

**DOI:** 10.1186/s12967-021-02874-7

**Published:** 2021-05-11

**Authors:** Yuchen Du, Qiuying Chen, Ying Fan, Jianfeng Zhu, Jiangnan He, Haidong Zou, Dazhen Sun, Bowen Xin, David Feng, Michael Fulham, Xiuying Wang, Lisheng Wang, Xun Xu

**Affiliations:** 1 Department of Automation, The Institute of Image Processing and Pattern Recognition, Shanghai Jiao Tong University (SJTU), 800 Dongchuan RD. Minhang District, Shanghai, 200240 People’s Republic of China; 2 Department of Preventative Ophthalmology, Shanghai Eye Diseases Prevention and Treatment Center, Shanghai Eye Hospital, No. 380 Kangding Road, Shanghai, 200040 China; 3 Department of Ophthalmology, Shanghai Key Laboratory of Ocular Fundus Diseases, Shanghai Engineering Center for Visual Science and Photo Medicine, Shanghai General Hospital, SJTU School of Medicine, Shanghai, China; 4 National Clinical Research Center for Eye Diseases, Shanghai, 20080 China; 5 Biomedical and Multimedia Information Technology Research Group, School of Computer Science, The University of Sydney, Sydney, NSW 2006 Australia; 6 Department of Molecular Imaging, Royal Prince Alfred Hospital and the University of Sydney, Sydney, Australia

## Correction to: J Transl Med (2021) 19:167 https://doi.org/10.1186/s12967-021-02818-1

Following publication of the original article [1], the authors identified an error in the author name of Xiuying Wang.

- The incorrect author name is: Xiuiyng Wang
- The correct author name is: Xiuying Wang

The author group has been updated above and the original article [1] has been corrected.
