# Supplementary material for: Automatic identification of myopic maculopathy related imaging features in optic disc region via machine learning methods
Source: J Transl Med. 2021 Apr 26;19:167. doi: 10.1186/s12967-021-02818-1 (PMC8074495; doi:10.1186/s12967-021-02818-1)
Supplement: Supplementary file 1 — Additional file 1: Supplementary Material. [file 12967_2021_2818_MOESM1_ESM.docx]

Supplementary Material

# The Setting of Random Forest Classifier

The random forest classifier was employed as our classification model in this research. We utilized RandomForestClassifier package from the sklearn library to implement our experiment. The parameters of our model were listed in **Table 1**. The parameter “n_estimators” was set to 500 to make the model more stable. For all the other parameters in the classifier, we followed standard parameter settings to avoid performance bias that might be imposed by different datasets.

# Further Exploration of the Variation from PDCA to MDCA

The variation of PDCA and MDCA was further explored by categorizing them into the same group and then validated their differences with regard to the new selected features.

Two additional experiments were designed in this section. Firstly, we categorized both PDCA and MDCA into not severe MM group (C0, C1, PDCA and MDCA were categorized into one group while C3 and C4 were categorized into another) and repeated the feature selection and classification experiments. Secondly, we categorized PDCA and MDCA into severe MM group (C0 and C1 were categorized into one group while PDCA, MDCA, C3 and C4 were categorized into another) and conducted the same experiment. The number of features we selected also followed the principle that called for a tradeoff between the classification performance and the refinement of the model.

The classification performances of both additional experiments had decreased comparing with categorizing PDCA and MDCA into two different groups, as shown in **Table 2**. Meanwhile, for each new selected feature from the additional experiments, we had calculated the mean values and standard deviation in each subclass. Among the features, four top ranked new selected features as well as clinic feature AreaPPA were selected to illuminate the distribution properties of subclasses. As shown in **Figure 1A**, the variation from PDCA to MDCA was larger than that from MDCA and C3. While in **Figure 1B**, again, the variation from PDCA to MDCA was larger than that from C1 and PDCA.

# Features Importance and Classification Performance with regard to Different Numbers of Selected Features

The classification performance changes with regard to models consisting of different numbers of the selected features were investigated in this section.

We chose the models consisting of 8, 14 and 19 features, corresponding to the local maximum values of the classification performance, respectively. The average AUC of the model consisting of 8 features was 0.9041. When the number of features arose to 14 and 19, the average AUC scores of the models increased to 0.9051 and 0.9069, respectively.

The selected features as well as the feature importance of the Random Forest models trained on 8, 14 and 19 features were shown in **Figure 2**, **Figure 3** and **Figure 4**, respectively. The 8 new image features overlapped among the three models, while the top three highest-ranked features (firstorder_Energy_R, firstorder_Entropy_B, Coarseness_NGTDM) remained consistently higher than the other features in different models. Regarding to the classification performance, the additional 6 more features contributed 0.001 (0.11%) gain on AUC score, while 11 more features contributed 0.0028 (0.31%) gain on AUC score, demonstrating the very limited performance improvements.

# Multiple Feature Selection Strategies Analysis

Mutual information scores and the student’s t test were employed to validate the features importance.

The eight new image features as well as the six clinic features were validated in this section, as shown in **Table 3**. All of the eight new image features had p values less than 0.001. The mutual information scores of the top three ranked features were higher than 0.42, indicating these features were valid.

**Table 1** The parameters of RandomForest model

| Model | Parameters | Values |
| --- | --- | --- |
| Random Forest Classifier | bootstrap | TRUE |
|  | class_weight | None |
|  | criterion | 'gini' |
|  | max_depth | None |
|  | max_features | 'auto' |
|  | max_leaf_nodes | None |
|  | min_impurity_decrease | 0 |
|  | min_impurity_split | None |
|  | min_samples_leaf | 1 |
|  | min_samples_split | 2 |
|  | min_weight_fraction_leaf | 0 |
|  | n_estimators | 500 |
|  | n_jobs | -1 |
|  | oob_score | FALSE |
|  | random_state | 0 |
|  | verbose | 0 |
|  | warm_start | FALSE |

**Table 2** Classification performance of different categorization principle

| Classification performance | Categorize PDCA and MDCA into different groups | Categorize PDCA and MDCA into severe MM group | Categorize PDCA and MDCA into not severe MM group |
| --- | --- | --- | --- |
| clinic features | 0.7925 | 0.7308 | 0.7185 |
| new image features | 0.8263 | 0.7743 | 0.7095 |
| Union features | 0.8358 | 0.7805 | 0.7206 |

**Table 3** Mutual information and student's t test of new image features and clinic features

| New image feature index | Scores of mutual information | P value | Clinic feature index | Scores of mutual information | P value |
| --- | --- | --- | --- | --- | --- |
| firstorder_Energy_R | 0.443309 | <0.001 | AreaPPA | 0.452549 | <0.001 |
| firstorder_Entropy_B | 0.426932 | <0.001 | Tilt | 0.229600 | <0.001 |
| Coarseness_NGTDM | 0.430595 | <0.001 | Dist_MD | 0.174367 | 0.08 |
| PPAweight_u20R | 0.380971 | <0.001 | AreaDisc | 0.175783 | 0.02 |
| glcm_shade_135 | 0.264918 | <0.001 | Angle_MD | 0.162486 | 0.42 |
| PPAweight_u30R | 0.369207 | <0.001 | Torsion | 0.208406 | 0.13 |
| fourier_Circularity_PPA | 0.275846 | <0.001 |  |  |  |
| DiscCurvature13 | 0.250297 | <0.001 |  |  |  |


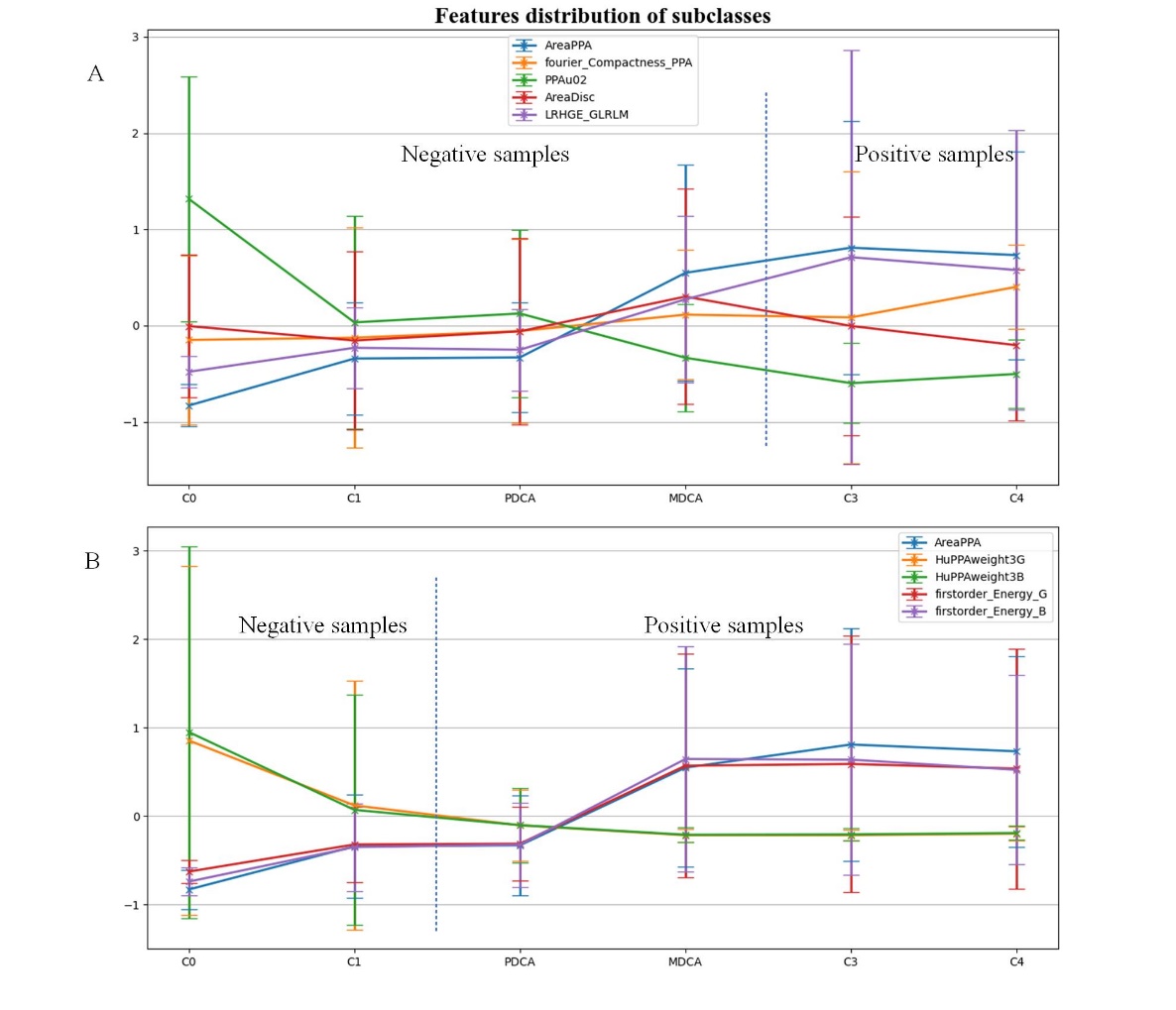


**Figure 1** The mean values and standard deviation values of features changed among subclasses. A) the distribution of the new selected features on subclasses when categorizing PDCA and MDCA into not severe MM group. B) the distribution of the new selected features on subclasses when categorizing PDCA and MDCA into severe MM group.


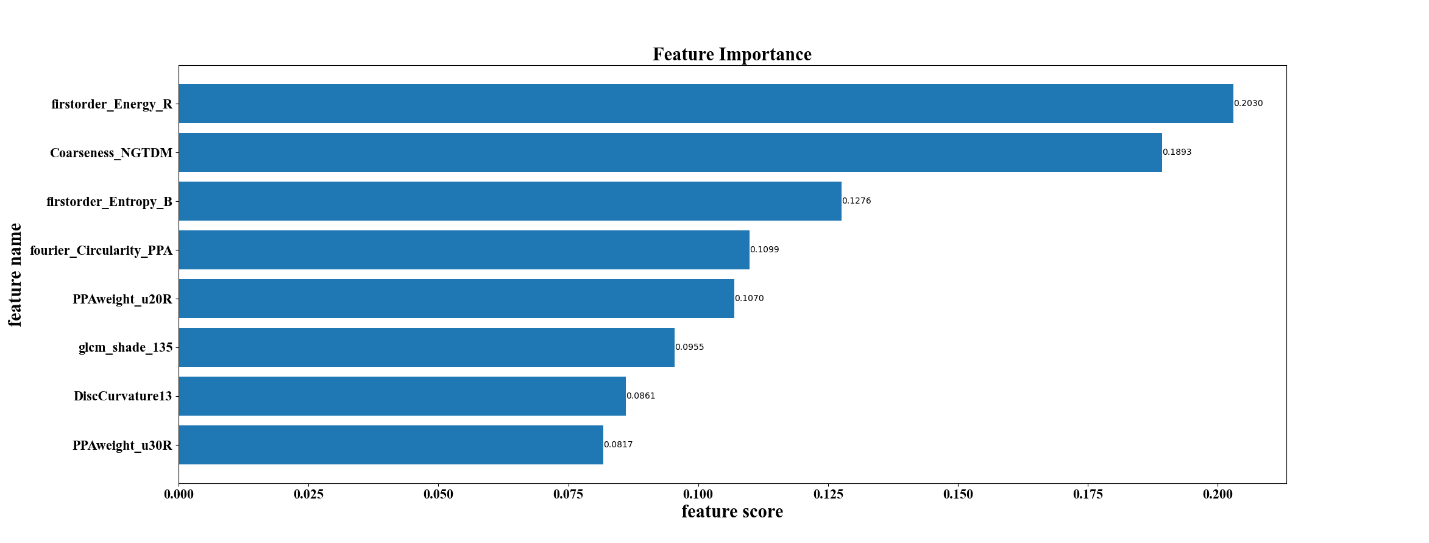


**Figure 2** Feature importance of RandomForest model consisting of 8 features


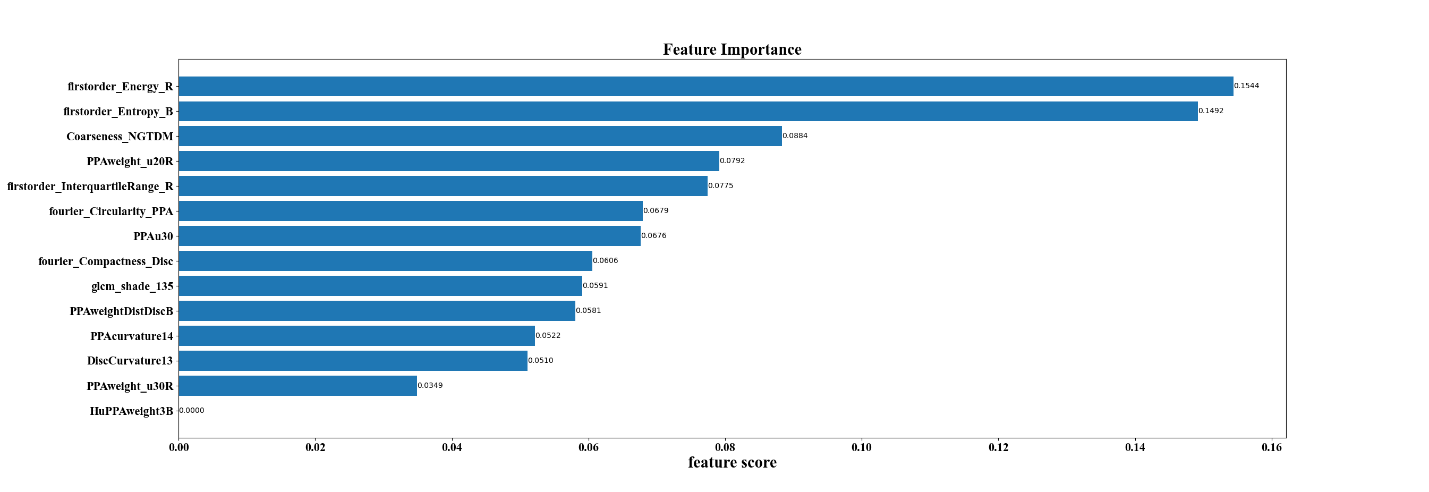


**Figure 3** Feature importance of RandomForest model consisting of 14 features


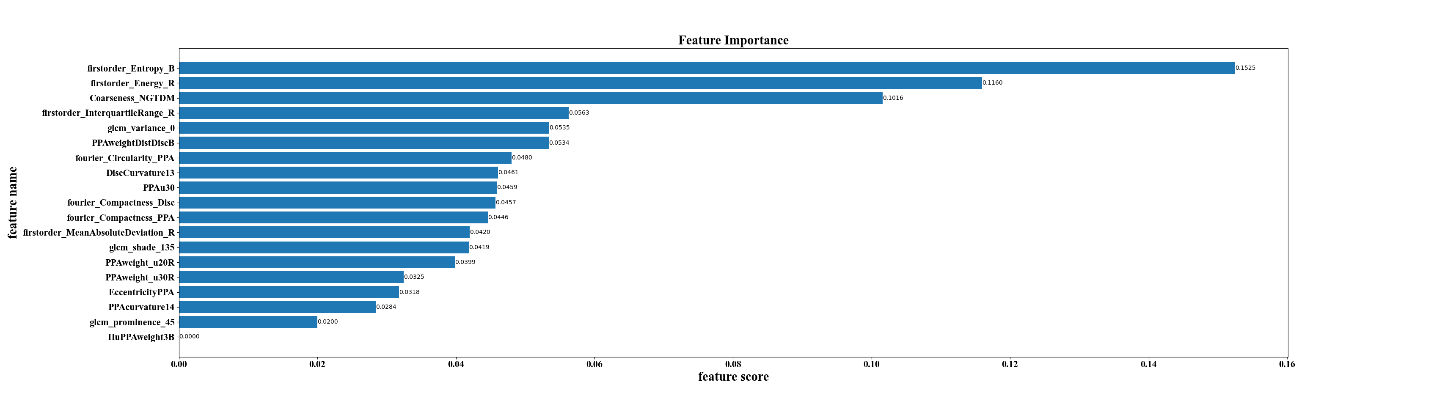


**Figure 4** Feature importance of RandomForest model consisting of 19 features
